# Generic and low dose antiretroviral therapy in adults and children: implication for scaling up treatment in resource limited settings

**DOI:** 10.1186/1742-6405-7-18

**Published:** 2010-06-23

**Authors:** Reshmie Ramautarsing, Jintanat Ananworanich

**Affiliations:** 1 The HIV Netherlands Australia Thailand Research Collaboration (HIVNAT), Bangkok, Thailand; 2 Centre for Poverty-related Communicable Diseases (CPCD), Department of Internal Medicine, Academic Medical Centre, University of Amsterdam, Amsterdam, the Netherlands; 3 The Southeast Asia Research Collaboration with Hawaii (SEARCH), Bangkok, Thailand; 4 Faculty of Medicine, Chulalongkorn University, Bangkok, Thailand

## Abstract

Although access to antiretroviral therapy (ART) for the treatment of HIV has increased during the last decade, many patients are still in need of treatment. With limited funds to provide ART to millions of patients worldwide, there is a need for alternative ways to scale up ART in resource limited settings. This review provides an overview of pharmacokinetic, safety and efficacy studies of generic and reduced dose ART. The production of generic ART has greatly influenced the decline in drug prices and the increased in ART access. Generic ART has good pharmacokinetic profile, safety and efficacy. Toxicity is however the main cause for ART discontinuation. Several dose reduction studies have shown adequate pharmacokinetic parameters and short term efficacy with reduced dose ART. Ethnicity may affect drug metabolism; several pharmacokinetic studies have confirmed higher plasma ART concentration in Asians. Randomized efficacy trial of reduced versus standard ART is warranted.

## Introduction

In 2008, an estimated 33.4 million adults and children were living with HIV worldwide [1], most of whom were from low and middle income countries, and 9.6 million people were in need of antiretroviral treatment (ART) [2]. However, 5.5 million people (58%) had no access to treatment. Even though the great majority of HIV infected people live in Sub-Saharan Africa, 4.7 million HIV infected people are living in Asia [1]. The ART coverage in East, South and South-East Asia was only 37% in 2008 [3]. Although this is an increase compared to the 29% in 2007, the scaling up of antiretroviral therapy is still slow. This review will focus on two important ways of achieving ART scale up in resource-limited settings: safe and effective generic ART, and dose reduction of ART.

### Generic Antiretroviral Therapy

In 2001 the World Health Organization (WHO) initiated the prequalification of priority medicines to make these available to millions of patients in need in resource-limited settings. In 2004, the U.S. Food and Drug Administration (FDA) launched a program to ensure that HIV patients being served by the President's Emergency Plan for AIDS Relief (PEPFAR) would receive safe, effective and quality manufactured ART. This new initiative included an expedited review process, and a strong encouragement for manufacturers worldwide to submit U.S. marketing applications for previously approved antiretroviral therapies, even if there was still a patent or exclusivity market protection for the product in the U.S. Currently, the FDA has given tentative approval to 107 generic antiretroviral drugs [4] which gives generic manufacturers the opportunity to produce safe, effective and good quality antiretroviral therapy combinations without having to face patent claims.

The introduction of generic fixed dose combination (FDC) antiretroviral therapy by companies in India and Thailand has significantly increased the access to treatment in many resource limited countries and is a major contributing factor to the unprecedented drop in ART prices. Between 2004 and 2008 the drug prices for first line regimens declined by 48%, and resulted in sustained scale up of treatment programs, transaction volume growth and competition between a growing number of drugs prequalified by the WHO. The decline in prices between 2004 and 2008 for second line treatment can also be attributed to the prequalification of the generic alternatives for abacavir (ABC), lopinaivir/ritonavir (LPV/r) and tenofovir (TDF) [3]. However, in 2009, the prices for second line regimens were still high in countries where few or no prequalified generic alternatives are available.

### Pharmacokinetics of generic ART

In a healthy volunteer study, the pharmacokinetic (PK) parameters of the generic FDC of d4T/3TC/NVP was compared to PK parameters of the three branded products, administered simultaneously [5]. Because this was a cross-over study, the patients were used as their own control. The generic FDC was proven to be bioequivalent to the administration of the three branded formulations of d4T, 3TC and NVP [5].

In a cross sectional study to evaluate the LPV minimum concentration (C_min_) in Thai HIV-1 infected adults using the Matrix LPV/r generic tablet version, it was found that patients had a median (IQR) LPV C_min _of 7.2 (5.8-8.3) mg/l, which was well above the LPV therapeutic level of 1.0 mg/l [6]. In another PK study from Thailand, the Matrix generic LPV/r was bioequivalent to the pediatric branded tablets (LPV/r 200/50 mg, Abbott) in adults with HIV infection [7]. This study utilized pediatric instead of adult branded tablets as Abbott has not marketed this product in Thailand in response to the Thai Government's compulsory licensing policy [6].

### Safety, efficacy and tolerability of generic ART in adults

In Thailand, the Government Pharmaceutical Organization (GPO) began producing several antiretroviral drugs in 1995, but it was not until 2002, when GPO produced its first FDC of stavudine (d4T), lamivudine (3TC) and nevirapine (NVP) (GPO-VIR-S^®^) that HIV in Thailand changed from a deadly disease into a manageable chronic disease [8]. In patients with advanced HIV infection (CD4 count of less than 100 cells/mm^3 ^at baseline), the GPO-VIR-S^® ^combination had good efficacy, with 63.7% of patients showing plasma HIV-RNA of less than 50 copies/ml after 48 weeks of treatment [8]. The median decline in plasma HIV RNA from baseline was 3.8 log_10 _copies/ml (range 0.2-2.4) at week 48, which is comparable to the results of the 2NN study, in which 65.4% of the patients on (branded) d4T/3TC/NVP achieved virological suppression after 48 weeks [9]. A second study assessing the efficacy of the GPO-VIR-S^® ^combination had a median follow up period of 15 weeks, during which 54% of the patients achieved virological suppression [10]. These results demonstrate the effectiveness of the generic FDC d4T/3TC/NVP.

The effectiveness and safety of the FDC of TDF/emtricitabine (FTC)/efavirenz (EFV) was illustrated in HIV infected adults in western India [11]. Both ART-naïve and -experienced patients showed excellent immunological and virological response and adherence. None of the patients in this study experienced clinical or immunological failure, and the median change in CD4 count after 12 months was +368 cells/mm^3 ^among the ART-naïve patients and +176 cells/mm^3 ^among the ART-experienced patients. Similarly, the virological response was high: 96% of all patients had plasma HIV-RNA less than 400 copies/ml after 6 months. The most common toxicity experienced was EFV related neuropsychiatric complaints; grades 1 and 2 in 16 patients, and 1 patient (0.7%) had to discontinue the regimen due to grade 4 neuropsychiatric toxicity. This rate is lower than those reported in developed countries [12,13]. However, the grades 3 and 4 renal toxicity was higher than in the published literature with 4 patients (2.8%) discontinuing the regimen [12,14]. This renal toxicity was likely from TDF and it was mainly found in patients with pre-existing renal disease.

However, the first line generic ART being used in resource-limited settings, mainly d4T-based regimens, have been associated with adverse events. HIV infected patients who had completed a minimum of 3 months of first line generic highly active antiretroviral therapy (HAART) in India were followed for a total of 6504 person years to assess the spectrum of adverse events [15]. The majority of patients (75%) were on a d4T-containing regimen and 53.4% developed at least one adverse event (most commonly rash 15.2%, peripheral neuropathy 9.0%, and anemia 5.4%) and 46.3% of these patients consequently changed or discontinued their regimen. Studies in developed countries have also identified toxicity as a major reason for regimen changes or discontinuation; a study from the United States reported that 47% of discontinuations were due to toxicity [16], and in the Swiss HIV Cohort study 46.6% of the treatment modifications in the first year after starting HAART were due to toxicity [17]. Sivadasan et al. however, reported a much higher rate: 68.1% of the changes in first line generic antiretroviral regimens in their cohort in South India were due to WHO grade 3 and 4 toxicity [18]. The most common toxicities were lactic acidosis in 20 (32.3%) patients, severe anemia in 16 (25.8%) patients and polyneuropathy in 12 (19.4%) of the patients. In this cohort, 76% of the patients were started on a d4T-containing regimen. Moreover, 70% of the patients had WHO clinical stage 3 or 4 before starting HAART. In the multivariate analysis, advanced HIV disease was one of the predictors for regimen change, together with current smoking, body mass index of more than 25 kg/m^2 ^and baseline elevated liver transaminases. More importantly to note is that lactic acidosis, severe anemia and polyneuropathy are all caused by the thymidine-analogue nucleoside reverse transcriptase inhibitors (NRTI) d4T and AZT. Current Western guidelines recommend the use of a TDF-based regimen for first line [19,20], and as a result d4T and AZT are used less and less in developed countries. The 2010 WHO guideline now recommends the use of TDF or AZT in the first line, and to avoid the use of d4T due to "the disfiguring, unpleasant and potentially life threatening toxicity of d4T" [21]. The high cost of TDF however, remains a barrier to the implementation of TDF as first line in resource limited settings. This underscores the need to make effective and safe generic TDF-based regimens for wide distribution in developing countries. In light of the current cuts in worldwide funding programs that provide antiretrovirals (ARVs) for millions of HIV patients in resource limited countries, it has been suggested to look into the potential of reducing the d4T dose in order to decrease toxicity, while maintaining virological efficacy [22]. This will be discussed in more detail below.

### Safety, efficacy and tolerability of generic ART in children

An estimated 3 million children are currently infected with HIV, and in 2008, only 38% of those children who were in need of HIV treatment had access to and were treated with ART [1]. Children are an extremely vulnerable group. Due to an immature immune system, the course of disease in children is extremely aggressive. Scaling up access to pediatric treatment has been slow, and there are a number of reasons for this, e.g. a lack of focus on HIV infected children by many governments, higher cost for pediatric formulations (50-90% higher than adult versions for branded products), a lack of ARV formulations for use in children, a lack of appropriate strength tablets, limited liquid formulations, a lack of pediatric labeling for many ARVs and difficulty gaining registration in many countries. Many studies have shown that children in resource limited settings respond as well to ART as children in resource rich settings [23]. Puthanakit et al. assessed the long term rates of viral suppression and immune recovery in 107 ART naïve Thai children with advanced HIV infection [24]. After four years of treatment 70% of the children had plasma HIV-RNA below 50 copies/ml, and the mean CD4-percentage increased from 5.3% at baseline to 26.6%, demonstrating the long term effectiveness of HAART in a resource limited setting.

An emerging problem however, is the development of first line treatment failure in children. Of the children enrolled in the Therapeutics, Research, Education and AIDS Training in Asia (TREAT Asia) program, 20% were on their second ARV regimen [25]. In China's National Pediatric ART Program 27.5% of the ART-naïve children and 62.5% of the ART experienced children showed resistance to one or more drugs after one year of treatment [26]. These numbers are worrisome, as evidence-based studies guiding the management of treatment failure in children are lacking, and the number of second line ARVs available for children, as well as the access to these medications, are limited [27]. For children who failed a 2NRTI plus a non-nucleoside reverse transcriptase inhibitor (NNRTI) regimen, a boosted protease inhibitor (PI) regimen is preferred [28]. Due to its high cost, the access to PIs is still limited for pediatric treatment. To address this issue, Puthanakit et al. assessed the LPV plasma concentrations in Thai HIV-infected children being treated with the adult tablet formulation of the Matrix generic LPV/r, and compared these to the LPV plasma concentrations during treatment with the branded soft gel capsule (SGC) formulation in the same patients [29]. The adult generic tablet, administered as the whole tablet, or in fractions, resulted in a median (IQR) LPV C_min _of 6.7 (5.0-9.9) mg/l, which was comparable to the LPV C_min _after treatment with the SGC, 7.3 (4.4-9.8) mg/l, *P *= 0.87. Importantly, 24% of the children had LPV C_min _higher than 10 mg/l, which is a particular concern; given that long term exposure to high concentrations of LPV may be a risk for dyslipidemia [30]. Two other studies in Thailand have also highlighted the issue of elevated PI plasma concentrations in Thai children. Bunupuradah et al. described relatively high plasma levels of saquinavir and LPV, and an increase in lipids after 96 weeks of double boosted PI (saquinavir/LPV/r) treatment in pre-treated HIV infected children [31]. Furthermore, Plipat et al. reported that a reduced dose of indinavir (IDV) boosted with ritonavir leads to adequate IDV plasma concentrations in pre-treated HIV infected children [32]. Further studies to assess the long-term safety and efficacy of reduced dose PIs and its potential to reduce toxicity and cost are needed.

### ART Dose reduction

The majority of the dose finding studies has been conducted in Caucasian men, and often relatively high ARV doses have been used to avoid sub-therapeutic levels. Evidence indicating that Asian patients have higher plasma concentrations for several ARVs compared to Caucasians is mounting. Genetic differences between ethnicities may be the primary cause for altered drug metabolism, and as a result, different PK parameters. Here we describe the dose reduction studies for different ARVs (Table 1).

**Table 1:** Summary of dose reduction for Antiretrovirals

Drug	Recommended dose	Reduced doses with supportive PK/short term efficacy data
**NRTIs**		

d4T [22,33]	30 mg BID	30 mg when > 60 kg 20 mg when < 60 kg

AZT [34,35]	300 mg BID	200 mg BID

**NNRTIs**		

EFV [39]	600 mg OD	400 mg OD

NVP when co-administered with rifampin [44]	Not recommended	400 mg BID

**PIs**		

IDV/r [45]	800/100 mg BID	400/100 mg BID

SQV/r [46-48]	1000/100 mg BID	1600 or 1500/100 mg BID

ATV [51,52]	400 mg OD or 300/100 mg OD	200/100 mg OD

LPV/r during 3^rd ^trimester of pregnancy [60]	600/150 mg BID	400/100 mg BID

NRTIs: nucleoside reverse transcriptase inhibitors. NNRTIs: non-nucleoside reverse transcriptase inhibitors. PIs: protease inhibitors. BID: twice daily. OD: once daily, PK: pharmacokinetic

### NRTIs

NRTI dose reductions are proven to be safe and effective. Dose reduction of d4T to 20 mg twice daily for patients with body weight less than 60 kg and 30 mg twice daily for patients with body weight more than 60 kg has been shown to be effective, with a more favorable toxicity profile [22,33]. Furthermore, AZT 300 mg twice daily, which is the recommended dosage, results in a 5-fold increases in plasma AZT concentrations in Thais [34]. Dose reduction from AZT 300 mg to 200 mg twice daily in Thais who weigh less than 60 kg resulted in comparable AZT plasma concentrations as AZT 300 mg twice daily in Caucasians with a mean weight of 74 kg [35].

### NNRTIs

#### Efavirenz (EFV)

Most of the Asian studies that assess the PK parameters of EFV have been conducted in patients taking rifampicin at the same time. EFV plasma concentrations can be reduced when co-administered with rifampicin [36] and in the past, an increase from EFV 600 mg to 800 mg once daily has been suggested when co-administering with rifampicin. A study in Thailand comparing EFV 600 mg with EFV 800 mg in patients using rifampicin showed that EFV plasma concentrations were similar [37], with excellent virological and immunological responses in both groups after 48 weeks [38] suggesting that Thai patients have sufficient EFV plasma concentrations even in the presence of rifampicin. Currently however, there is no consensus about the need to increase EFV dosage during rifampicin treatment; the WHO and United States Department of Health and Human Services guidelines do not recommend a dose increase, whereas the European AIDS Clinical Society does [19-21]. In Thai patients not taking rifampicin, EFV plasma concentrations were compared between EFV 400 mg once daily and EFV 600 mg once daily in the same patients. The 400 mg once daily dose resulted in, significantly lower, but still adequate plasma concentrations compared to the 600 mg once daily dosing, again demonstrating that Thai patients generally have higher plasma concentrations of several ARVs and that dose reduction does not compromise the efficacy [39].

EFV plasma concentrations are highly variable, and this variability may largely be depended on genetic variation of the gene that encodes the CYP450-2B6 isoenzyme. This isoenzyme is responsible for the 8-hydroxylation of EFV and for about 90% of its clearance. Individuals with a heterozygous or homozygous 516G > T polymorphism have significantly higher EFV concentrations compared to individuals with the wild-type polymorphism [40]. Puthanakit et al. demonstrated that the children in their cohort had adequate EFV plasma concentrations [41] and there was a strong correlation between the CYP2B6-516G > T polymorphism and EFV plasma concentrations.

#### Nevirapine (NVP)

Rifampicin reduces the nevirapine area under the curve (AUC) by 20 to 58% [19,42] and Western guidelines recommend not to use this combination in HIV-tuberculosis co-infected patients [19,20]. However, NVP is still the NNTRI of choice in many developing countries, since it is widely used in the majority of FDCs. Thai patients receiving a NVP-based HAART regimen with rifampin had their mean plasma NVP concentrations compared to Thai patients receiving NVP-based HAART without rifampin [43]. Even though the plasma concentrations in the rifampin group were considerably lower than in the group without rifampin, the great majority (86%) had adequate NVP plasma concentrations. In another study from Thailand, patients using rifampicin were randomized to NVP 400 mg per day or NVP 600 mg per day [44]. The results indicated that 400 mg per day had a similar efficacy as 600 mg, but the patients receiving 600 mg per day were more likely to experience adverse events related to NVP. Therefore, dose increase during rifampin treatment is not recommended in Thai patients [44].

### Protease Inhibitors - PIs

#### Indinavir (IDV)

The recommended dose for IDV boosted with Ritonavir (RTV) is 800/100 mg twice daily. However, the use of IDV is highly associated with renal toxicity, especially in patients with IDV AUC of higher than 60 h*mg/l. In Thai HIV infected patients, a reduced dose of IDV/r 400/100 mg twice daily is effective and well tolerated [45]. In that study, the median (IQR) of IDV C_min _was 0.17 (0.12-0.30) mg/l and 80% of the patients had IDV concentrations above the therapeutic level of 0.10 mg/l.

#### Saquinavir (SQV)

SQV/r is dosed as 1000/100 mg twice daily. A study comparing the PK parameters of three different dosages in Thai patients (SQV/r 1600/100 mg once daily, 1000/100 mg twice daily and 2000/100 mg once daily) found that both 1000/100 mg twice daily and 2000/100 mg once daily resulted in a higher AUC and C_min _compared to 1600/100 mg once daily [46]. However, the mean C_min _of all three were higher than the recommended C_min _of 0.1 mg/l. Furthermore, SQV/r 1600/100 mg once daily was shown to have strong antiviral efficacy when used with an NRTI backbone in Thai HIV-infected patients [47,48]. In a study comparing SQV 600 mg with SQV 1000 mg twice daily, co-administered with either LPV/r 400/100 twice daily or 266/66 mg twice daily in Thai ARV naïve patients, SQV dose reduction to 600 mg resulted in adequate PK parameters, with a higher SQV AUC when co-administered with LPV/r 400/100 mg compared to LPV/r 266/66 mg [49]. With accumulating pharmacokinetic data indicating dose reduction is safe and effective, SQV is now recommended at 1500 (or 1600 mg) once daily, co-administered with RTV 100 mg once daily in the treatment of ART-naive Thais.

In children who failed an NRTI/NNRTI based HAART regimen, a double boosted PI regimen (SQV and LPV/r) was demonstrated to have strong virological and immunological response after 48 weeks and 96 weeks [31,50]. However, as mentioned before, the C_min _of both PIs were higher than the therapeutic concentrations, resulting in elevated lipids and suggesting further exploration of the possibility of dose reduction of PI in children.

#### Atazanavir (ATV)

ATV can be dosed once daily. It has a low pill burden and a good toxicity profile, and is one of the preferred PI choices in guidelines [19,20]. ATV levels are boosted by RTV. The standard once daily dose of ATV is 400 mg without RTV boosting in treatment naïve patients and 300 mg ATV with 100 mg RTV boosting in treatment experienced patients. The dose reduction of ATV/r from 300/100 mg once daily to 200/100 mg once daily in Thai adults has been investigated. The lower 200/100 mg once daily dosing showed lower, but still adequate PK parameters, and none of the patients had an ATV C_min _lower than the therapeutic level of 0.15 mg/l [51]. After dose reduction, there was a significant reduction in serum bilirubin. In a smaller study, also in Thailand, 14 patients who were treated with ATV/r at 200/100 mg once daily had good virological and immunological responses after 68 weeks [52].

#### Lopinavir (LPV)

In Asia, LPV/r is the PI that is most used as part of second line regimens. The recommended dose is LPV/r 400/100 mg twice daily. The original formulation, the soft gel capsule (SGC, 133.3/33.3 mg) needs to be taken with food, and requires refrigerated storage. More recently, a tablet formulation has been developed (200/50 mg), and it has better bioavailability and no food or refrigerated storage requirements. A lower dose of LPV/r at 266/66 mg twice daily used together with SQV displayed adequate LPV PK parameters in adults [49]. Similarly in a pediatric study, Thai HIV-infected, PI-naïve children were treated with either the WHO recommended dose of LPV/r or 70% of the standard dose, with 2 NRTI backbone [53]. The PK parameters of LPV and RTV were not significantly different in both groups, and after 48 weeks the safety and efficacy were excellent.

Because an earlier study showed evidence for high C_min _in Thais using generic LPV/r [6], a subsequent study by the same group of investigators evaluated the PK of twice daily LPV/r reduced dose (200/50 mg twice daily) as well as the PK of the generic versus the branded LPV/r. They found that generic LPV/r had an equivalent PK profile as the branded product, however, the reduced LPV/r dose of 200/50 mg twice daily had inadequate LPV PK parameters [7]. This was most likely because a reduction of the RTV dose by 50% was inadequate to boost LPV plasma concentrations. In contrast to IDV, SQV and ATV, LPV levels appear to be highly dependent on the amount of RTV [54]. Reduced dose of LPV combined with a higher dose of RTV (e.g. LPV200mg/RTV100mg) could possibly result in adequate LPV levels and should be further investigated.

In pregnant women, the metabolism of several drugs is altered due to a change in the physiology, particularly the expansion of intravascular volume and the induction of the hepatic CYP 450 system that leads to higher rates of drug metabolism [55,56]. Both can lead to insufficient drug levels, especially in the third trimester [57]. An increase in the LPV/r dose is recommended in the third trimester [58], but guidelines have not yet adapted this recommendation, and state that data are not yet conclusive as to the optimal dose during pregnancy [59]. A PK study was done in Thai HIV-infected pregnant women who were using the standard dose of 400/100 mg twice daily [60]. PK curves were recorded at gestational age 20 weeks (GA20), GA 33 and 12 weeks post-partum (12PP). Twelve women recorded both the GA33 and the 12PP curve; the mean LPV AUC was significantly lower at GA33 compared to 12PP. At GA 33, 95% of the women had sufficient LPV plasma concentration above 1.0 mg/l and at 12PP all women had LPV plasma concentration above 1.0 mg/l, indicating that Thai HIV-infected pregnant women do not require a LPV/r dose increase during the third trimester of pregnancy. This highlights the needs to conduct studies in different ethnic groups as guidelines developed based on Caucasian PK data cannot be extrapolated to other ethnicities.

## Conclusions

The data summarized in this review underscores the need to explore alternative options to scale up ART for resource limited settings, particularly safe and effective generic ART, and dose reduction of ART. Current evidence support the bioequivalence, safety and efficacy of generic ART compared to branded products. More effort is needed to scale up generic FDCs using drugs with favorable toxicity profiles such as TDF-based regimens for first and second line regimens. Data on ART dose reduction, mainly from small PK studies in Thailand, suggest that reduced doses of ART do not compromise PK parameters, and short term safety and efficacy. This warrants larger randomized studies to evaluate the efficacy of reduced dose ART. Such effort is underway for low dose EFV 400 mg once daily as first line ART (ENCORE study, clinicaltrials.gov NCT01011413).

## Competing interests

The authors declare that they have no competing interests.

## Authors' contributions

RR reviewed the literature and drafted the manuscript. JA gave scientific input and edited the manuscript. All authors read and approved the final manuscript.
